# Upregulation of HMGB1-TLR4 inflammatory pathway in focal cortical dysplasia type II

**DOI:** 10.1186/s12974-018-1078-8

**Published:** 2018-01-30

**Authors:** Zhongbin Zhang, Qingzhu Liu, Ming Liu, Hui Wang, Ying Dong, Taoyun Ji, Xiaoyan Liu, Yuwu Jiang, Lixin Cai, Ye Wu

**Affiliations:** 1 Department of Pediatrics, Peking University First Hospital, No.1 Xi’an Men Street, West District, Beijing, 100034 China; 2 Children Epilepsy Center, Peking University First Hospital, No.1 Xi’an Men Street, West District, Beijing, 100034 China; 3 Department of Pathology, Peking University First Hospital, No.8 Xishiku Street, West District, Beijing, 100034 China

**Keywords:** HMGB1-TLR4, Upregulation, Inflammation, FCD type II, Neurons, Astrocytes

## Abstract

**Background:**

We attempted to determine whether the inflammatory pathway HMGB1-TLR4 and the downstream pro-inflammatory cytokines is upregulated in focal cortical dysplasia (FCD) type II and whether there is a correlation between the TLR4 upregulation and disease duration or frequency of epileptic seizures.

**Methods:**

FCD type II and peri-FCD paired tissues resected from eight children with refractory epilepsy were collected. Through real-time qPCR, Western blot, and co-immunoprecipitation, we examined the differences between FCD lesions and peri-FCD tissues with respect to mRNA expression, protein expression, and protein interaction in HMGB1-TLR4 pathway biomarker and downstream pro-inflammatory factors in whole brain tissue. Then, we used immunofluorescence to examine the difference between FCD lesions and peri-FCD tissues with respect to protein expression and intracellular distribution of HMGB1-TLR4 pathway biomarker in neurons, astrocytes, and oligodendrocytes. Correlation between level of TLR4 expression and disease duration or frequency of epileptic seizures in patients was also analyzed.

**Results:**

The protein expression levels of TLR4, cytoplasm HMGB1, TLR4/MyD88 complex, ubiquitination of TRAF6, p-IKK, p-IκB-α, p-NF-κB p65, and IL-1β and TNF-α in lesion tissues were significantly higher than those in peri-FCD controls. Total mRNA expression levels of TLR4, IL-1β, and TNF-α in lesion tissues were significantly higher than those in peri-FCD controls, but HMGB1 had no significant change. In neurons and astrocytes inside the lesions, the expression of TLR4 protein was significantly higher than that in peri-FCD tissues, and HMGB1 was mainly expressed in the cytoplasm, while expressed in the nuclei in peri-FCD tissues. But in oligodendrocytes, there was no upregulation of HMGB1-TLR4 pathway in both lesions and peri-FCD tissues. We did not identify the correlation between the level of TLR4 activation and disease duration or frequency of epileptic seizures.

**Conclusion:**

The HMGB1-TLR4 pathway was upregulated in the neurons and astrocytes inside FCD type II lesions, which led to an increase in the release of downstream pro-inflammatory cytokines. Correlation between the level of TLR4 activation and duration or frequency of epileptic seizures was not identified.

## Background

In recent years, a growing body of evidence has shown that inflammatory response plays a role in epileptogenesis [1–4]. The Toll-like receptor 4 (TLR4) signaling pathway is the most studied pathway. TLR4, as a pattern recognition receptor, can promote innate immune response and mediate inflammatory responses to lipopolysaccharide [5]. In central nervous system (CNS), TLR4 is expressed in astrocytes, oligodendrocytes, microglia, and neurons. TLR4 expression is low in the resting state. In some pathological conditions, TLR4 expression in the cytoplasm increases and TLR4 proteins are transferred to the cell membrane [5]. High mobility group box-1 (HMGB1) is one of the important endogenous activators of TLR4. After TLR4 activation, the downstream pathway is activated primarily through a MyD88-dependent pathway. An activated TLR4 recruits MyD88, thereby causing ubiquitination of TRAF6 and NF-κB inflammatory pathway activation. An activated NF-κB moves into the nucleus and promotes the transcription of pro-inflammatory cytokine IL-1β and TNF-α [5]. Previous studies showed that the TLR4 pathway plays a role in ischemic brain injury, brain trauma, CNS infection, neurodegenerative disease, and brain autoimmune inflammation [5–7], whereas only a preliminary understanding of the role of TLR4 in epileptogenesis has been obtained in recent years [8–10]. Focal cortical dysplasia (FCD) is one of the major causes of refractory epilepsy and is the most common histopathological type in children with lesional epilepsy [11, 12]. In this study, we collected FCD type II and peri-FCD paired samples of surgically operated tissues from eight children with refractory epilepsy. We examined TLR4 and its endogenous agonist HMGB1 and detected the expression of TLR4/MyD88 complex, ubiquitination of TRAF6, phosphorylation of IKK, phosphorylation of IκB-α, phosphorylation of NF-κB p65, and pro-inflammatory cytokines IL-1β and TNF-α in the downstream pathways. We attempted to determine whether the HMGB1-TLR4 pathway is upregulated in FCD type II lesions and to provide further evidence on the role of inflammatory response in epileptogenesis.

## Methods

### Selection of patients

The patients met the following inclusion criteria: (1) diagnosed as having refractory epilepsy according to the criteria defined by the International League Against Epilepsy; (2) focal cortical dysplasia was suggested in brain magnetic resonance imaging (MRI): focal changes in the cortical thickness, signal increase (mainly on T2FLAIR and T2WI sequences), gray-white matter blurring, and the presence of a “transmantle” sign; (3) other etiologies potentially causing epilepsy were excluded; (4) lesions were confirmed to be FCD type II (a or b) in postoperative pathology; (5) peripheral zone of resected lesions were microscopically confirmed without dysplastic neurons and balloon cells; and (6) the size of lesions was large enough to satisfy the need of both clinical pathological examination and the research. Forty patients diagnosed with FCD type II and underwent lesion resection were collected from January 2016 to April 2017 in the Children Epilepsy Center, Peking University First Hospital. Of these patients, 17 met the inclusion criteria. We randomly selected eight cases, including three cases of FCD type IIa and five FCD type IIb.

The following clinical data were collected: (1) demographic data; (2) information associated with epilepsy, particularly age of onset, seizure type, seizure frequency, status epilepticus, and antiepileptic drugs; and (3) information associated with lesion resection, particularly age of operation, location of FCD, and pathological type of FCD. Detailed clinical data are shown in Table 1.

**Table 1:** The clinical data of eight children with FCD type II

Case ID	1	2	3	4	5	6	7	8
Gender	Female	Male	Male	Male	Female	Female	Male	Female
Age of onset	6 months	1 year	40 days	3 years	7 months	4 months	6 months	1 year and 10 months
Seizure type	FS	FS	FS	FS	FS	Spasm	FS, atypical absence	FS
Seizure frequency	10/day	1–2/day	2–10/day	1/week	1–4/day	3–4/day	2–4/day	3–10+/day
Status epilepticus	None	None	None	None	None	None	EPC	None
Antiepileptic drugs	VPA, LEV, TPM, VGB	OXC, LTG, PB, VPA, TPM, CZP, LEV	VPA, OXC, TPM, PB, LEV, VGB	LEV, CBZ, LTG, VPA, TPM	VPA, OXC, PB, LTG, LEV, TPM	VPA, TPM, OXC	VPA, LEV, CZP, OXC	VPA, OXC, TPM, LTG, NZP
Age of operation	1 year and 2 months	7 years and 3 months	2 years	4 years and 2 months	3 years and 6 months	1 year and 2 months	7 years and 3 months	13 years
Location of FCD	Left temporal	Right parietal	Left parietal	Left frontal	Left central	Right frontal	Right frontal	Right temporal
Pathological type of FCD	FCD IIa	FCD IIa	FCD IIa	FCD IIb	FCD IIb	FCD IIb	FCD IIb	FCD IIb

*VPA* valproate, *CBZ* carbamazepine, *OXC* oxcarbazepine, *PB* phenobarbital, *TPM* topiramate, *LEV* levetiracetam, *LTG* lamotrigine, *CZP* clonazepam, *NZP* nitrazepam, *VGB* vigabatrin, *FS* focal seizures

### Acquisition of brain tissue samples

During the operation, brain tissue sample inside the FCD and in the peri-lesional zone were obtained respectively; both were 1 cm^3^ in size. Each sample was divided into two parts. One part was stored in liquid nitrogen and prepared for protein and total RNA, whereas the other part was fixed in 4% buffered formalin and prepared for the immunofluorescence test.

### Ethics and informed consent

The study was approved by the clinical research ethics committee of Peking University First Hospital. Written informed consents were obtained from the parents.

### Detection of HMGB1-TLR4 pathway in FCD and peri-FCD

#### Detection of HMGB1-TLR4 pathway in a tissue sample

##### Western blot analysis

For detecting TLR4 and HMGB1 protein expression, membrane and cytoplasmic proteins were extracted by Pierce® Mem-PER Plus Membrane Protein Extraction Kit (#89842, Thermo Scientific). And for other biomarkers, total proteins were extracted by RIPA buffer. Antibodies to HMGB1 (rabbit monoclone, 1:1000, Abcam), TLR4 (mouse monoclone, 1:500, Santa Cruz), IKK-β (monoclone rabbit, 1:500, CST), phospho-IKKα/β (rabbit monoclone, 1:500, CST), IκB-α (mouse monoclone, 1:500, CST), phospho-IκB-α (rabbit monoclone, 1:500, CST), NF-κB p65 (rabbit monoclone, 1:500, CST), phospho-NF-κB p65 (rabbit monoclone, 1:500, CST), IL-1β (rabbit monoclone, 1:500, CST), and TNF-α (rabbit monoclone, 1:500, CST) were used in a routine Western blot analysis on whole tissue sample. The expression of β-actin (monoclonal mouse, 1:1000) was used as loading control. All experiments were performed independently for three times.

##### Co-immunoprecipitation

Co-immunoprecipitation (Co-IP) was performed using a Thermo Scientific Pierce Co-IP kit (#26149) following the manufacturer’s protocol. Briefly, the antibodies of TLR4 (10 μg/mL, Santa Cruz) and TRAF6 (10 μg/mL, CST) were first immobilized for 2 h using an AminoLink Plus coupling resin. The resin was then washed and incubated with arterial lysate overnight. After incubation, the resin was washed, and the protein was eluted with an elution buffer. The samples were analyzed by Western blotting as previously described. Anti-TLR4 (1:500, Santa Cruz), anti-MyD88 (1:500, Abcam), anti-TRAF6 (1:1000, CST), and anti-K63 polyubiquitin chain (1:500, CST) were used for the analysis. All experiments were performed independently for three times.

##### Real-time quantitative PCR

Real-time PCR was utilized for detecting the mRNA expression levels of TLR4, HMGB1, IL-1β, and TNF-α. The sequences of the forward (F) and reverse (R) primers are listed 5′ to 3′ as follows: HMGB1 (F) AAGCACCCAGATGCTTCAGT, (R) TCCGCTTTTGCCATATCTTC; TLR4 (F) AATCCCCTGAGGCATTTAGG, (R) AAACTCTGGATGGGGTTTCC; IL-1β (F) GGAACCCGTGTCTTCCTAAAG, (R) CTGACTTGGCAGAGGACAAAG; TNF-α (F) GGCGTGGAGCTGAGAGATA, (R) CAGCCTTGGCCCTTGAAGA; GAPDH (F) GGAGCGAGATCCCTCCAAAAT, (R) GGCTGTTGTCATACTTCTCATGG. The cycling conditions were performed as follows: initial denaturation at 95 °C for 5 min, followed by 45 cycles of denaturation at 95 °C for 15 s, annealing at 60 °C for 5 s and extension at 72 °C for 10 s. All experiments were performed independently for three times. Amplified GAPDH was used as endogenous control gene to analyze the real-time PCR data of the target genes and data analyzed using the 2^−ΔΔCt^ method [13].

#### Detection of HMGB1-TLR4 pathway in glia and neurons

Immunofluorescence double staining was carried out to detect the expression and distribution of HMGB1 and TLR4 in neurons, astrocytes, and oligodendrocytes. The brain tissue was fixed in 10% buffered formalin for 24 h. In all cases, including lesion and peripheral zone, a representative formalin-fixed, paraffin-embedded tissue block was performed. Paraffin-embedded tissue was sectioned at 4 μm and mounted on precoated glass slides. The tissue sections were blocked with 5% normal goat serum for 1 h at 37 °C. After that, the sections were incubated with the following primary antibodies: HMGB1 (rabbit monoclone, 1:200; mouse monoclone 1:50 Abcam), TLR4 (mouse monoclone, 1:100, Abcam), GFAP (mouse monoclone, 1:200, CST; rabbit monoclone, 1:200, CST), NeuN (rabbit monoclone, 1:500, Abcam; mouse monoclone, 1:500, Abcam), and Olig2 (rabbit polyclone, 1:100, CST) at 4 °C for overnight. On the following day, after rinsing with phosphate-buffered saline (PBS), sections were incubated with Alexa Fluor 568 and Alexa Fluor 488 (anti-rabbit IgG or anti-mouse IgG; 1:500) for 2 h at room temperature. The cell nuclei were labeled using Hoechst 33342.

Quantitative analysis was performed for the percentage of double-labeled positive cells. Briefly, five representative non-overlapping fields of the tissues were captured and digitized with a laser scanning confocal microscope (Olympus). We counted the total number of neurons (NeuN-positive), astrocytes (GFAP-positive), and oligodendrocytes (Olig2-positive) in each field. HMGB1, TLR4-positive in neurons, astrocytes, and oligodendrocytes were subsequently counted, and the percentage of TLR4-positive cells marked specific marker and the percentage of HMGB1-specific markers/Hoechst colocalization were calculated.

#### Correlation between TLR4 expression and clinical variables

After comparing FCD and peri-FCD of each patient, we investigated the correlation between the TLR4 expression levels in the lesions and clinical variables, including disease duration of epilepsy and seizure frequency. The disease duration of epilepsy was calculated by subtracting age of onset from age of operation. Seizure frequency was defined as the average number of seizures per day during the disease course. All experiments were performed independently for three times.

#### Statistical analysis

Statistical analysis was performed with SPSS 20.0. All data were expressed as mean ± SEM of the mean. Western blot, real-time PCR, and co-immunoprecipitation analyses between FCD and peri-FCD in the same case were performed using paired-sample *t* test. The correlation between TLR4 expression level and clinical variables was assessed using the Spearman’s rank correlation test. The immunofluorescent staining results were analyzed using the nonparametric Wilcoxon rank-sum test. A *P* value of 0.05 was considered statistically significant.

## Results

### Upregulation of HMGB1-TLR4 pathway in FCD II whole tissue samples

**Fig. 1:**
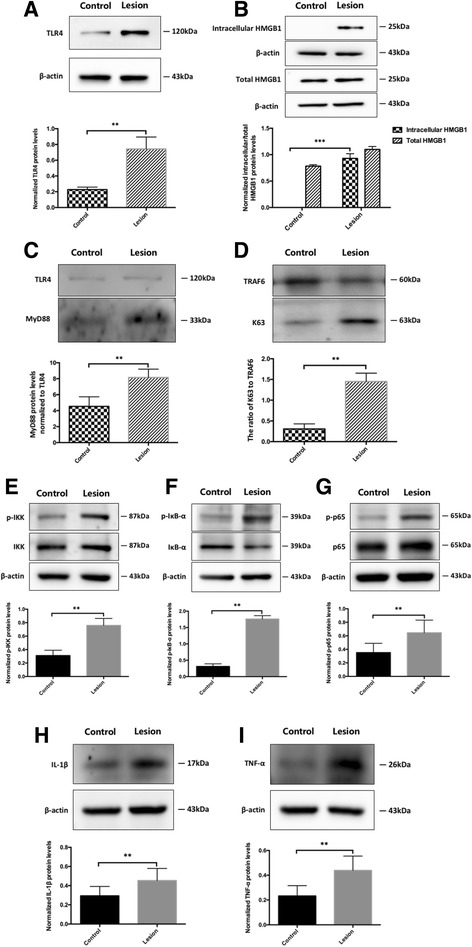
The protein expression of HMGB1-TLR4 pathway in FCD II whole brain tissue samples. Western blot was performed for detecting the biomarker protein expression of HMGB1-TLR4 pathway. **a** TLR4 protein expression in FCD lesion tissue was higher than that in peri-FCD tissue. **b** HMGB1 protein expression in the cytoplasm inside the FCD brain tissue was significantly higher than that in the peri-FCD tissue, but total expression had no significant difference between FCD and peri-FCD tissue. **c** MyD88 protein expression levels normalized to TLR4 in FCD lesion were higher than those in peri-FCD. **d** The expression ratio of K63 polyubiquitin chain to TRAF6 was increased in FCD lesion tissue, compared to that in peri-FCD tissue. **e**–**g** Phosphorylation levels of IKK-β, IκB-α, and NF-κB p65 in lesion tissue were higher than those in peri-FCD. **h**, **i** The expression levels of IL-1β and TNF-α were increased in FCD lesion. β-actin was used as a loading control. The results are expressed as the ratio of biomarkers to β-actin. The values are the mean ± SEM (*n* = 8) (**P < 0.01 and ***P < 0.001)

To detect the protein levels of TLR4 and intracellular/total HMGB1 in FCD and peri-FCD brain tissue, Western blot analysis was performed after extracting membrane, cytoplasmic, and total proteins. The results showed that the protein expression levels of TLR4 in FCD lesion tissue were significantly increased, compared to those in peri-FCD tissue (0.743 ± 0.152 vs. 0.227 ± 0.031; *n* = 8, *P* < 0.01; Fig. 1a). And HMGB1 protein expression in the cytoplasm inside the FCD brain tissue was significantly higher than that in the peri-FCD tissue (0.929 ± 0.089 vs. 0; *P* < 0.001; Fig. 1b), but total expression had no significant difference between FCD and peri-FCD tissue (1.099 ± 0.057 vs. 0.783 ± 0.028; *P* > 0.05; Fig. 1b), which suggested that HMGB1 was released from the nucleus into the cytoplasm and upregulated TLR4 expression in FCD lesion tissue.

Moreover, to explore the downstream of TLR4 pathway, TLR4/MyD88 complex, and ubiquitination of TRAF6 in the FCD and peri-FCD brain tissues, we performed co-immunoprecipitation and Western blot analysis. The results showed that the MyD88 protein expression levels normalized to TLR4 in FCD lesion were higher than that in peri-FCD (8.151 ± 1.042 vs. 4.528 ± 1.212; *n* = 8, *P* < 0.01; Fig. 1c), suggesting that MyD88 was recruited and formed TLR4/MyD88 complex in FCD lesion. And in lesion tissue, the expression ratio of K63 polyubiquitin to TRAF6 was increased, compared to that in peri-FCD tissue (1.449 ± 0.201 vs. 0.305 ± 0.124, *P* < 0.01; Fig. 1d), indicating that ubiquitination of TRAF6 was increased in FCD lesion. These results demonstrated that the downstream of TLR4 pathway also was activated in FCD lesion.

Furthermore, to investigate whether NF-κB inflammatory pathway, downstream of TLR4 pathway, also was activated, phosphorylation levels of IKK, IκB-α, and NF-κB as well as expression levels of the two cytokines IL-1β and TNF-α were examined by Western blot. We observed that phosphorylation levels of IKK (0.759 ± 0.104 vs. 0.311 ± 0.080; *n* = 8, *P* < 0.01; Fig. 1e), IκB-α (1.759 ± 0.104 vs. 0.311 ± 0.079; *P* < 0.01; Fig. 1f), and NF-κB p65 (0.646 ± 0.186 vs. 0.352 ± 0.138; *P* < 0.01; Fig. 1g) in lesion tissue were higher than those in peri-FCD, and the expression levels of IL-1β (0.452 ± 0.127 vs. 0.294 ± 0.098; *P* < 0.01; Fig. 1h) and TNF-α (0.439 ± 0.116 vs. 0.233 ± 0.083; *P* < 0.01; Fig. 1i) were increased in FCD lesion, which suggested that NF-κB pathway in lesion tissue was activated, and promote the expression levels of IL-1β and TNF-α.

**Fig. 2:**
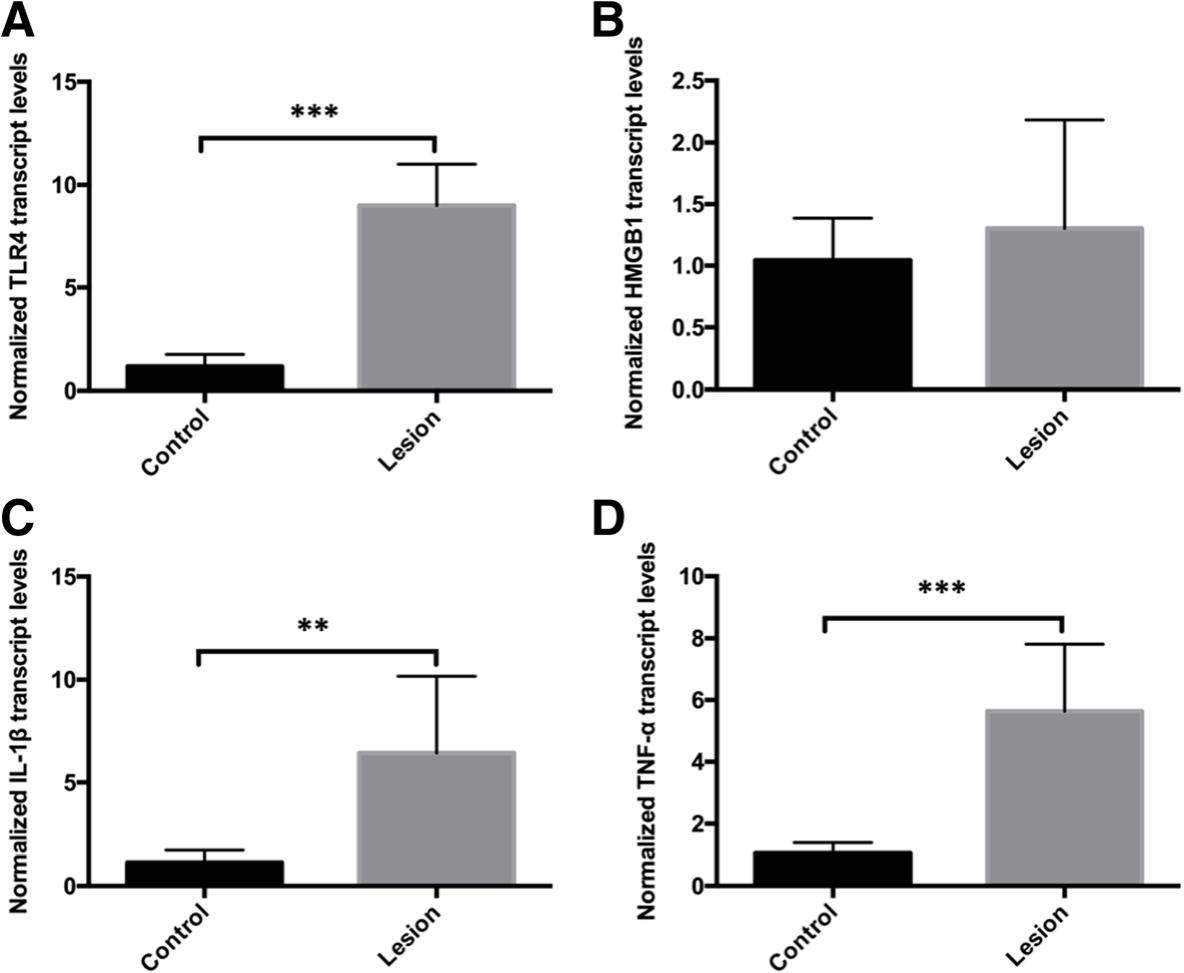
The mRNA expression of HMGB1-TLR4 pathway in FCD II whole tissue samples. Real-time PCR was performed for detecting mRNA expression levels of TLR4, HMGB1, and two pro-inflammatory cytokines IL-1β and TNF-α. **a**, **c**, **d** The mRNA expression levels of TLR4, IL-1β, and TNF-α in FCD brain tissues were significantly higher than those in the peri-FCD tissue. **b** No significant difference between FCD and peri-FCD was observed with respect to the mRNA expression of HMGB1. The values are the mean ± SEM (*n* = 8) (**P < 0.01 and ***P < 0.001)

To explore whether the mRNA expression levels of TLR4, HMGB1, and two pro-inflammatory cytokines IL-1β and TNF-α were changed in FCD lesion tissue, real-time PCR was utilized. The results indicated that there were significant increasing mRNA expression levels of TLR4 (8.999 ± 2.009 vs. 1.160 ± 0.585; *n* = 8, *P* < 0.001; Fig. 2a), IL-1β (6.428 ± 3.745 vs. 1.129 ± 0.586; *P* < 0.01; Fig. 2c), and TNF-α (5.641 ± 2.168 vs. 1.049 ± 0.341; *P* < 0.001; Fig. 2d) in the FCD brain tissues, compared with those in the peri-FCD, but no significant difference between FCD and peri-FCD was observed with respect to the mRNA expression of HMGB1 (1.303 ± 0.878 vs. 1.044 ± 0.343; *P* > 0.05; Fig. 2b). Combined with results of Western blot, we found that the expression of TLR4, IL-1β, and TNF-α had a significant increase.

### HMGB1-TLR4 pathway was upregulated in the neurons and astrocytes but not in the oligodendrocytes in FCD type II lesion tissues

To determine in which cell types of FCD lesion there is abnormal upregulation of HMGB1-TLR4 pathway, double staining was performed using anti-TLR4 and anti-HMGB1 antibodies, as well as antibodies specific for neurons (NeuN), astrocytes (GFAP), and oligodendrocytes (Olig2). The cell nuclei were labeled using Hoechst.

#### Neurons

**Fig. 3:**
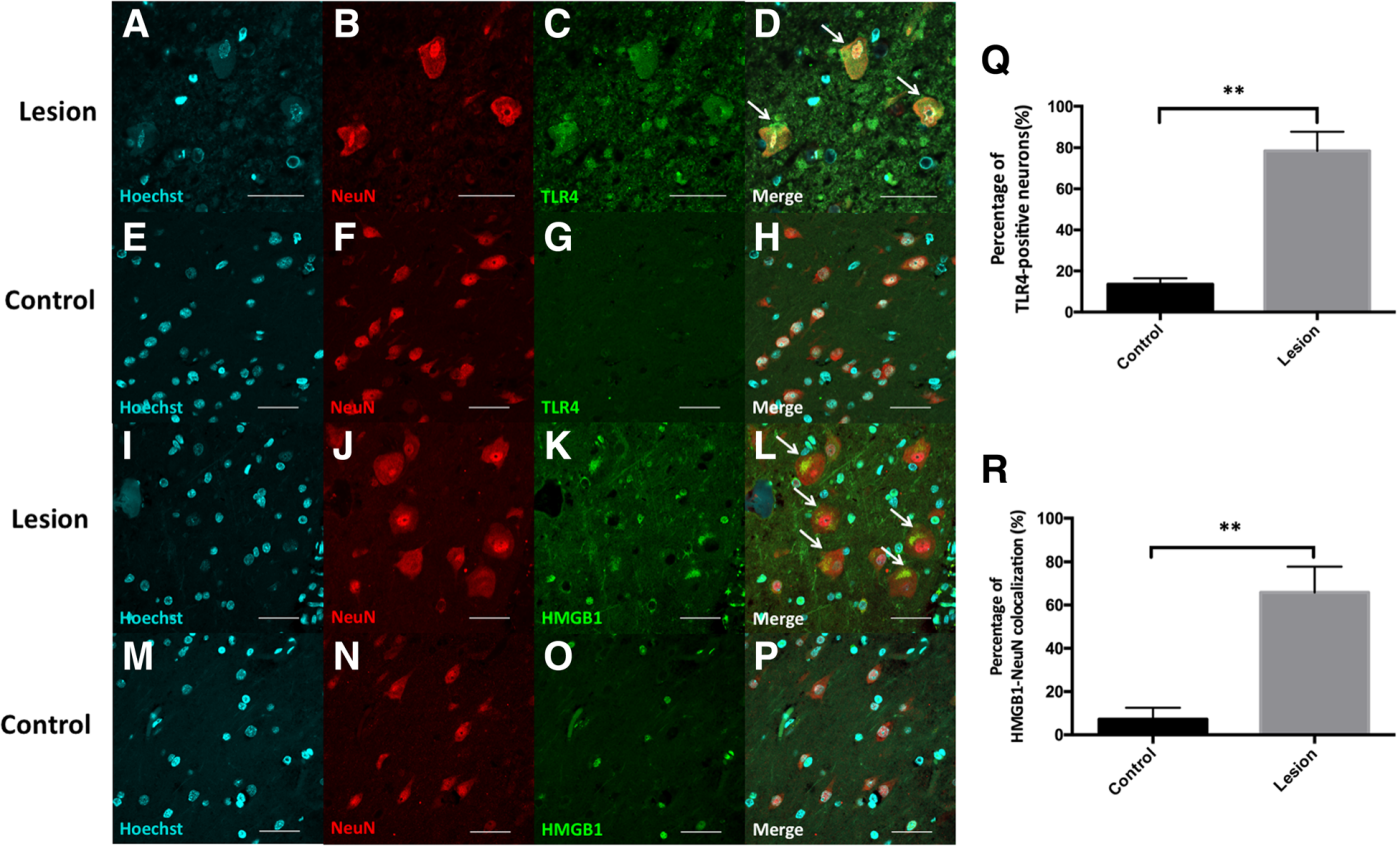
The expression and distribution of TLR4 and HMGB1 in neurons of FCD lesion and control. Immunofluorescence double staining for the expression and neuronal localization of TLR4 and HMGB1. TLR4 immunoreactivity (in green) in FCD lesion tissue was detected in neurons (as indicated by arrows in **d**) which were visualized by the neuronal marker NeuN (in red) (**a**–**d**), but not in control tissue (**e**–**h**). The percentage of TLR4-postive neurons in FCD and peri-FCD tissue was presented (**q**). HMGB1 immunoreactivity (in green) was mainly colocalized with NeuN in lesion area (**i**–**l**; as indicated by arrows in **l**), but with Hoechst 33342 (in cyan) in peri-FCD area (**m**–**p**). The percentage of HMGB1-NeuN colocalization in lesion and control area was presented (**r**). Scale bars = 40 μm. The values are the mean ± SEM (*n* = 8) (**P < 0.01)

The results showed that TLR4 immunoreactivity was colocalized with the neuronal marker NeuN in FCD lesion tissue (Fig. 3a–d), but not in peri-FCD tissue (Fig. 3e–h). And the percentage of TLR4-positive neurons in FCD lesion tissue was significantly higher than that in peri-FCD tissue (78.9 ± 9.3% vs. 12.5 ± 2.9%; *n* = 8, *P* < 0.01; Fig. 3q), which suggested that the expression of TLR4 was upregulated in the neurons of FCD lesion. Moreover, we also found HMGB1 was mainly colocalized with NeuN in lesion area (Fig. 3i–l), but with Hoechst in peri-FCD area (Fig. 3m–p). The percentage of HMGB1-NeuN colocalization in lesion area was higher than that in peri-FCD area (65.5 ± 11.9% vs. 7.4 ± 5.3%; *P* < 0.01; Fig. 3r), which indicated that HMGB1 was translocated from nucleus to cytoplasm in FCD lesion. These results demonstrated that HMGB1-TLR4 pathway was abnormally upregulated in the neurons of the FCD II brain tissue.

#### Astrocytes

**Fig. 4:**
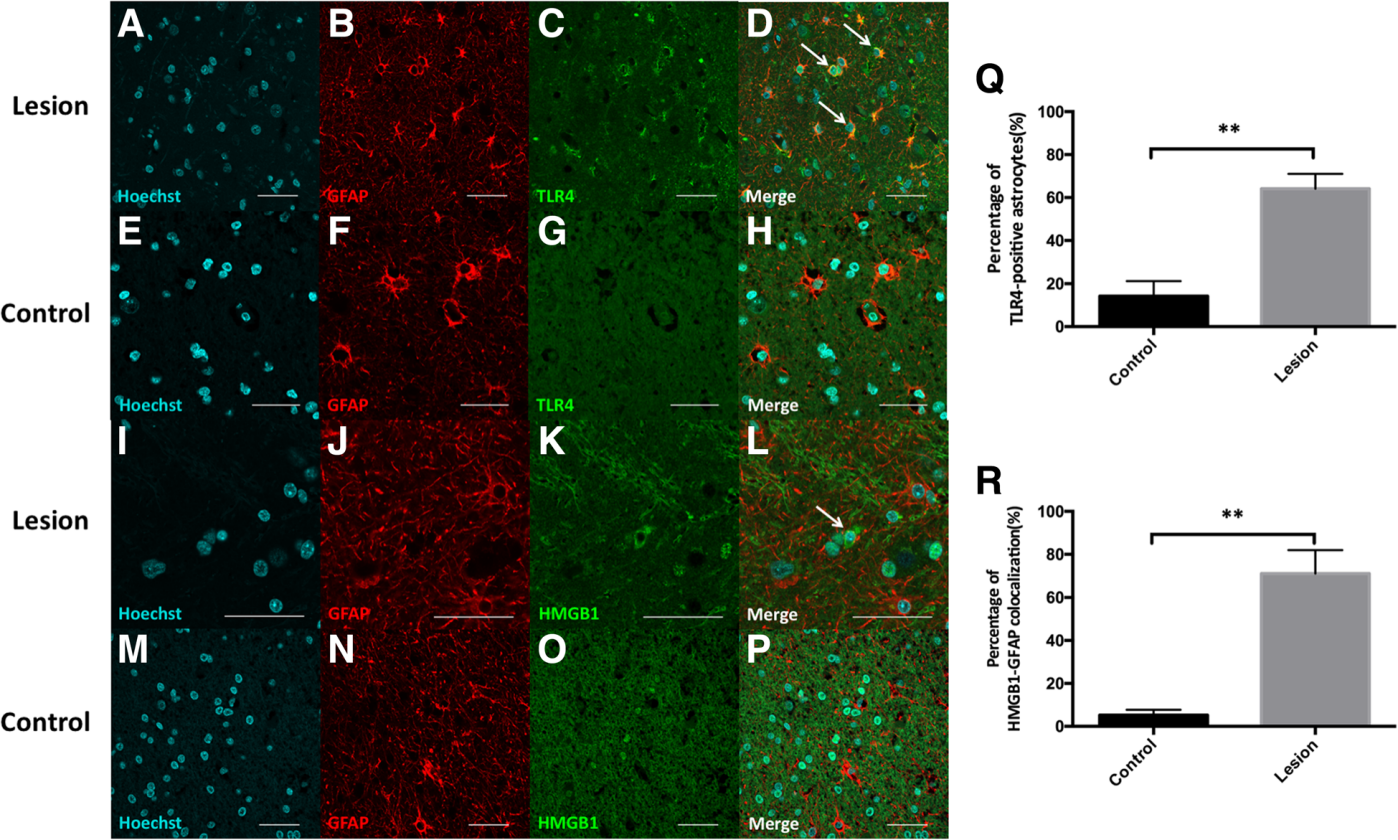
The expression and distribution of TLR4 and HMGB1 in astrocytes of FCD lesion and control. Immunofluorescence double staining for the expression and astrocytic localization of TLR4 and HMGB1. TLR4 immunoreactivity (in green) in FCD lesion tissue was detected in astrocytes (as indicated by arrows in **d**) which were visualized by the astrocytic marker GFAP (in red) (**a**–**d**), but not in control tissue (**e**–**h**). The percentage of TLR4-postive astrocytes in FCD and peri-FCD tissue was presented (**q**). HMGB1 (in green) was expressed in the astrocyte cytoplasm of the lesion area (**i**–**l**; as indicated by arrows in **l**), but with Hoechst 33342 (in cyan) in peri-FCD area (**m**–**p**). The percentage of HMGB1-GFAP colocalization in lesion and control area was presented (**r**). Scale bars = 40 μm. The values are the mean ± SEM (*n* = 8) (**P < 0.01)

We found that the TLR4-GFAP colocalization was increased in FCD lesion tissue, compared with peri-FCD lesion (Fig. 4a–h). And the percentage of TLR4-positive astrocytes in lesion tissue was significantly higher than that in control tissue (62.5 ± 7.8% vs. 13.9 ± 8.1%; *n* = 8, *P* < 0.01; Fig. 4q), which suggested that there is also upregulation of TLR4 in astrocytes of FCD lesion. And HMGB1 was expressed in the astrocyte cytoplasm of the lesion area (Fig. 4i–l), but in nucleus of peri-FCD area (Fig. 4m–p), and the percentage of HMGB1-GFAP colocalization in lesion area was higher than that in peri-FCD area (68.9 ± 12.3% vs. 5.1 ± 2.4%; *P* < 0.01; Fig. 4r). These data presented indicated that HMGB1-TLR4 pathway was also abnormally upregulated in the astrocytes of the FCD lesion tissue.

#### Oligodendrocytes

**Fig. 5:**
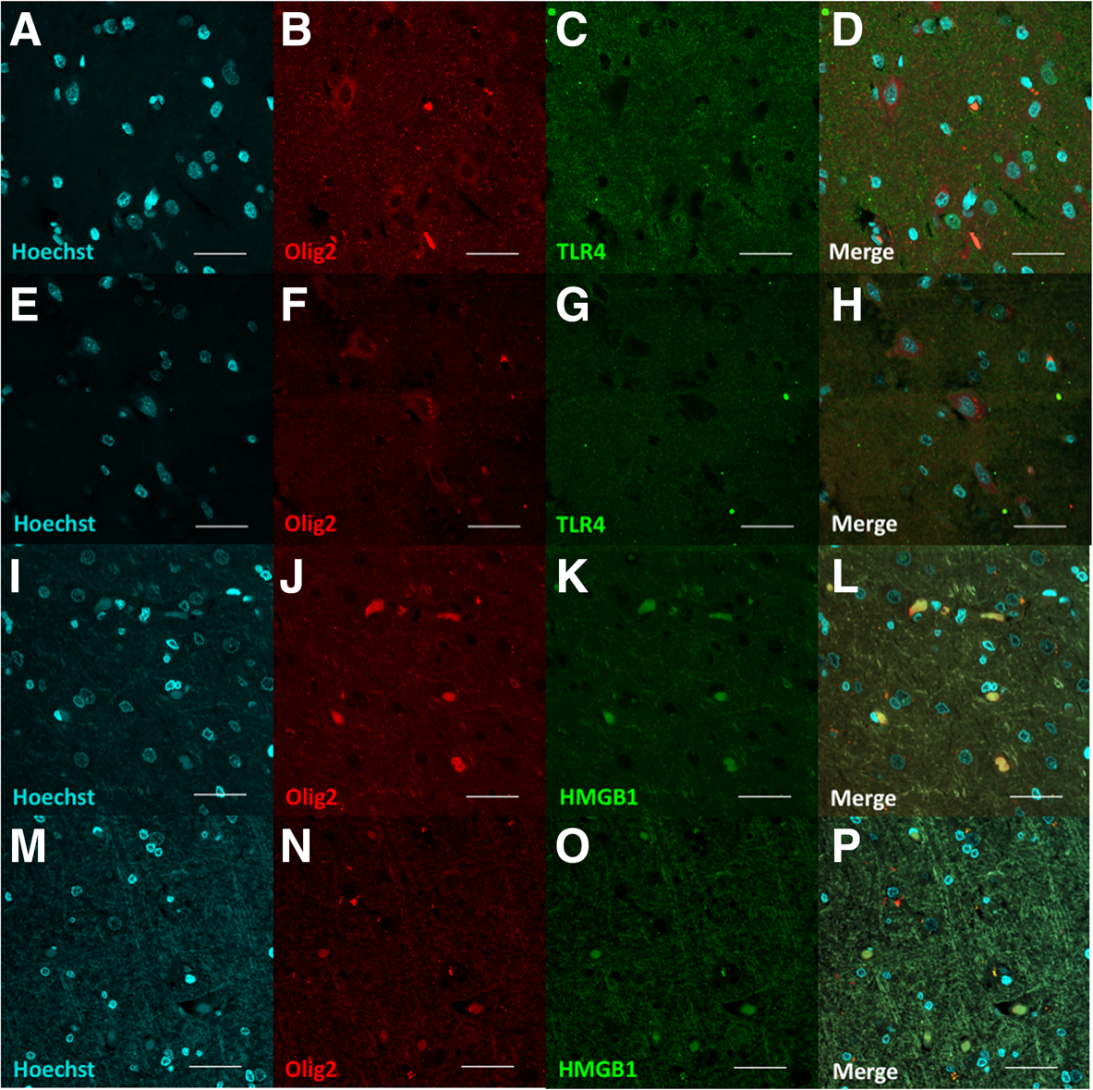
The expression and distribution of TLR4 and HMGB1 in oligodendrocytes of FCD lesion and control. Immunofluorescence double staining for the expression and oligodendrocytes localization of TLR4 and HMGB1. TLR4 immunoreactivity (in green) was not detected in oligodendrocytes which were visualized by the oligodendrocytic marker Olig2 (in red) in both of FCD and peri-FCD tissue (**a**–**h**). HMGB1 was mainly colocalized with Hoechst 33342 in oligodendrocytes of both FCD and peri-FCD tissue (**i**–**p**). Scale bars = 40 μm

The results showed that no TLR4-Olig2 colocalization TLR4 was observed in both of FCD and peri-FCD tissue (Fig. 5a–h), indicating low expression of TLR4 in oligodendrocytes of FCD lesion. HMGB1 was mainly expressed in the nuclei of FCD and peri-FCD tissue (Fig. 5i–p). These results suggested that no upregulation of HMGB1-TLR4 pathway occurred in the oligodendrocytes of the FCD brain tissue.

### Level of TLR4 upregulation was not correlated with disease duration of epilepsy or frequency of seizures

**Fig. 6:**
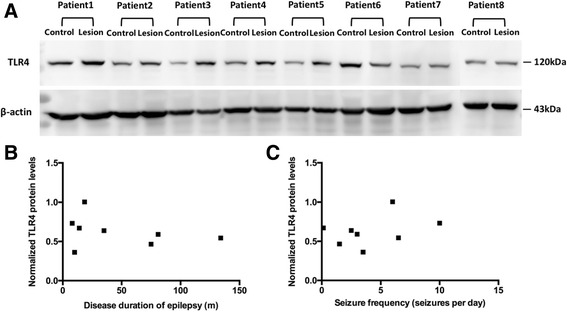
No correlation between TLR4 expression and clinical variables. **a** Western blot was performed for detecting protein expression levels of TLR4 between lesion and control among eight patients. **b**, **c** Scatter plot showed that there was no significant correlation between TLR4 expression and disease duration of epilepsy (month) (**b**) or seizure frequency (seizures per day) (**c**) in FCD II

To investigate whether there is a correlation between the TLR4 expression levels in the lesions and clinical variables, we examined TLR4 protein expression of each patient using Western blot and assessed the correlation between level of TLR4 upregulation and clinical variables, including disease duration of epilepsy and seizure frequency using the Spearman’s rank correlation test. The results showed that no significant association was observed between the level of TLR4 upregulation in FCD lesions and disease duration of epilepsy (*r* = − 0.333, *P* > 0.05; Fig. 6b) or seizure frequency (*r* = 0.214, *P* > 0.05; Fig. 6c).

## Discussion

Few previous studies suggested an upregulation of HMGB1-TLR4 pathways in epileptic brain tissues [8–10]. As an important pattern recognition receptor in the innate immune response, TLR4 is mainly expressed in the astrocytes, oligodendrocytes, microglia, and neurons in the brain [5]. In the resting state, TLR4 has low expression level in the brain, although it can be elevated in some pathological conditions (infection and tissue damage). TLR4 can be activated by exogenous pathogen-associated molecular patterns, such as lipopolysaccharide and endogenous damage-related molecular patterns, including HMGB1. And activated TLR4 can subsequently activate the downstream NF-κB pathway and promote the synthesis and release of IL-1β and TNFα. These processes lead to inflammatory response. In the resected lesion brain tissue of adult hippocampal sclerosis, Maroso et al. [8] showed that the expression level of TLR4 in the astrocytes and neurons was higher than that in normal tissues, and the expression of HMGB1 in astrocytes was increased in the cytoplasm, suggesting that HMGB1 in astrocytes was released from the nucleus to the cytoplasm. Zurolo et al. [9] found that in the lesion tissue of focal cortical dysplasia, cortical tubers of tuberous sclerosis, and ganglion glioma, TLR4 expression in the neurons and astrocytes is higher than that in normal human autopsy tissues and HMGB1 is significantly expressed in astrocyte cytoplasm. In this study, we used the surgically resected brain tissues form epileptic children with FCD II and compared them with the peripheral tissues from the same children. We found that TLR4 endogenous agonist HMGB1 was released from the nucleus into the cytoplasm. TLR4, TLR4/MyD88 complex, and ubiquitinated TRAF6 expression were upregulated, and the downstream NF-κB inflammatory pathway was activated, thereby increasing the expression of pro-inflammatory cytokines IL-1β and TNFα. Our findings provided a strong evidence for the upregulation of the HMGB1-TLR4 pathway in the epilepsy lesion, and this upregulation led to the release of downstream pro-inflammatory factors.

How was TLR4 pathway upregulated in epilepsy? Although TLR4 is involved in pathogen recognition and activated during infections, the HMGB1-TLR4 axis might underlie chronic epilepsy in scenarios without frank immune activation or infection. HMGB1 is a nonhistone chromosome-binding protein that is abundantly present in the nuclei of eukaryotic cells. When cells are damaged or stressed, HMGB1 is released from the nuclei of the injured or necrotic cells to the cytoplasm and extracellular membrane [14]. The release of HMGB1 can be considered a dangerous signal from the damaged or stressed cells to alert tissue of the status of acute or persistent damage. In this study, we confirmed that HMGB1 is released from the nucleus to the cytoplasm and extracellular membrane in neurons and astrocytes inside FCD lesions. HMGB1 was released to the extracellular membrane, and it interacted with TLR4 and activated the endogenous immune mechanism of neurons or glial cells and TLR4-associated inflammatory response. In this study, HMGB1 remained expressed in the nucleus of the peri-FCD brain tissue, suggesting that TLR4 may have been upregulated by the release of extracellular HMGB1 in chronic injury caused by repeated epileptic activities in FCD lesion. Furthermore, we also found that the transcript levels of HMGB1 had no significant difference between FCD and peri-FCD tissue. Combined with the results that the protein expression of intracellular HMGB1 in lesion were higher than those in peri-FCD but the total one had no change in lesion, these suggested that repeated epileptic electrical activities had an effect on the cytosolic translocation of HMGB1, but no express levels.

The upregulation of HMGB1-TLR4 pathway in epileptic lesions was likely to be the result of repeated epileptic electrical activities, but whether the upregulation of the pathway will further promote epileptogenesis in turn? No direct evidence on the vicious circle between epileptogenesis and inflammation was reported. In patients with tuberous sclerosis, Zurolo et al. [9] found that TLR4 and HMGB1 were expressed in the giant embryonic cells of cortical tubers. This finding suggested that the activation of HMGB1-TLR4 pathway may occur earlier than epileptic episodes, which mean that the induction of these inflammatory signaling pathways may be inherent in the occurrence and development of the disease, and its continued activation may be one of the reasons causing the occurrence of late epilepsy. Maroso et al. [8] found that, in the acute and chronic epilepsy model of adult mice, injecting HMGB1 into the hippocampus of mice significantly increased the duration of seizures and shortened seizure latency, whereas injecting HMGB1 antagonist (BoxA) or TLR4 antagonist (Lps-Rs and Cyp) significantly inhibited the occurrence of acute and chronic seizures. This finding suggested that the upregulation of HMGB1-TLR4 pathway likely promotes the occurrence of epilepsy. In addition, the upregulation of HMGB1-TLR4 pathway occurred in the astrocytes of the FCD brain tissue. In recent years, the role of astrocytes in the occurrence and development of epilepsy has been studied [15, 16]. Astrocytes, in addition to being an important regulator of the brain’s microenvironment, have been shown to possess the function of innate immune cells. Reactive astrocytes are the source and target of multiple inflammatory factors, such as IL-1β, IL-6, and TNF-α [17]. In 2017, Shen et al. [18] found that the postnatal activation of TLR4 in the astrocytes of mice can promote excitatory synaptogenesis in hippocampal neurons. Therefore, we hypothesized that the upregulation of HMGB1-TLR4 and its downstream pathway not only might be the result of repeated epileptic discharge but also promote the occurrence of epilepsy, that is, inflammation and epilepsy were in reciprocal causation, leading to a vicious circle.

Furthermore, we also found that phosphorylation of IKK, IκB-α, and NF-κB as well as expression of two pro-inflammatory cytokines IL-1β and TNF-α in FCD lesion tissue were significantly higher than those in peri-FCD tissue, which suggested that NF-κB inflammatory pathway was activated in FCD lesion. Based on previous studies, the classical NF-κB activation pathway can be mediated through activation of a variety of cell surface receptors, including Toll-like receptors, IL-1 receptor, and TNF receptor, in response to pro-inflammatory mediators like LPS, IL-1, and TNF [19]. And activated NF-κB translocate from cytoplasm to nucleus and promote the transcription of pro-inflammatory cytokines like IL-1β and TNF-α, thus form the positive feedback [19]. So, in this study, activated NF-κB inflammatory pathway in FCD lesion was not only caused by upregulation of HMGB1-TLR4 pathway, but could also be a result of cytokine receptor activation.

In our study, we did not found significant association between the level of TLR4 upregulation in FCD lesions and disease duration of epilepsy or seizure frequency. Boer et al. [20] analyzed a possible correlation between the number of microglia (HLA-DR-positive) and macrophage (CD68-positive) within the dysplastic area and different clinical variables. The result showed that the number of microglia in FCD had a significant correlation with the duration of epilepsy and preoperative seizure frequency. This study suggested that the level of inflammatory response was likely related to clinical variables of epilepsy. We considered that the reason we did not find the correlation in our study is that TLR4 expression in our study was performed in a cross-sectional time, but the level of HMGB1-TLR4 pathway expression might be dynamically changed in the disease duration of epilepsy. Recent findings have shown dynamic changes in HMGB1 and its isoforms in the brain and blood of animals exposed to acute brain injuries and undergoing epileptogenesis [21].

## Conclusions

Our data demonstrated that the HMGB1-TLR4 pathway was upregulated in the neurons and astrocytes inside FCD type II lesions, and the upregulation of HMGB1-TLR4 led to an increase in the release of downstream pro-inflammatory cytokines. Correlation between the level of TLR4 activation and duration or frequency of epileptic seizures was not identified. According to our results presented, it was unclear whether the upregulation of the inflammatory pathway was involved in epileptogenesis or whether it was a consequence of seizures. But based on previous studies, we hypothesized that abnormal upregulation of inflammatory pathway was not only a causation but also a consequence of epileptic seizures, which lead to a vicious circle. We will explore evidence for proving reciprocal causation between inflammation and epilepsy in our future research.

## Abbreviations

Co-IP: Co-immunoprecipitation
FCD: Focal cortical dysplasia
GFAP: Glial fibrillary acidic protein
HMGB1: High mobility group box-1
MRI: Magnetic resonance imaging
PBS: Phosphate-buffered saline
TLR4: Toll-like receptor 4
